# Global abundance of short tandem repeats is non-random in rodents and primates

**DOI:** 10.1186/s12863-022-01092-4

**Published:** 2022-11-03

**Authors:** Masoud Arabfard, Mahmood Salesi, Yazdan Hassani Nourian, Iman Arabipour, AliMohammad Ali Maddi, Kaveh Kavousi, Mina Ohadi

**Affiliations:** 1 Chemical Injuries Research Center, Systems Biology and Poisonings Institute, Baqiyatallah University of Medical Sciences, Tehran, Iran; 2 Department of Biotechnology, Science and Research Branch, Islamic Azad University, Tehran, Iran; 3 Laboratory of Complex Biological Systems and Bioinformatics (CBB), Department of Bioinformatics, Institute of Biochemistry and Biophysics (IBB), University of Tehran, Tehran, Iran; 4 Iranian Research Center on Aging, University of Social Welfare and Rehabilitation Sciences, Tehran, Iran

**Keywords:** Global, Short tandem repeat, Abundance, Non-random, Rodent, Primate, Hierarchical clustering

## Abstract

**Background:**

While of predominant abundance across vertebrate genomes and significant biological implications, the relevance of short tandem repeats (STRs) (also known as microsatellites) to speciation remains largely elusive and attributed to random coincidence for the most part. Here we collected data on the whole-genome abundance of mono-, di-, and trinucleotide STRs in nine species, encompassing rodents and primates, including rat, mouse, olive baboon, gelada, macaque, gorilla, chimpanzee, bonobo, and human. The collected data were used to analyze hierarchical clustering of the STR abundances in the selected species.

**Results:**

We found massive differential STR abundances between the rodent and primate orders. In addition, while numerous STRs had random abundance across the nine selected species, the global abundance conformed to three consistent < clusters>, as follows: <rat, mouse>, <gelada, macaque, olive baboon>, and <gorilla, chimpanzee, bonobo, human>, which coincided with the phylogenetic distances of the selected species (p < 4E-05). Exceptionally, in the trinucleotide STR compartment, human was significantly distant from all other species.

**Conclusion:**

Based on hierarchical clustering, we propose that the global abundance of STRs is non-random in rodents and primates, and probably had a determining impact on the speciation of the two orders. We also propose the STRs and STR lengths, which predominantly conformed to the phylogeny of the selected species, exemplified by (t)10, (ct)6, and (taa4). Phylogenetic and experimental platforms are warranted to further examine the observed patterns and the biological mechanisms associated with those STRs.

## Introduction

Speciation is the evolutionary process by which populations evolve to become distinct species. Several models and theories have been proposed for this highly complicated process, including gene regulatory networks, community ecology, and mating preferences (for a review see [1]). Natural selection may be considered a major outcome associated with, and linking the above propositions. With an exceptionally high degree of polymorphism and plasticity, short tandem repeats (STRs) (also known as microsatellites/simple sequence repeats) may be a spectacular source of variation required for speciation and evolution [2–6]. The impact of STRs on speciation is supported by their various functional implications in gene expression, alternative splicing, and translation [4, 7–13].

STRs are a source of rapid and continuous morphological evolution[14], for example, in the evolution of facial length in mammals[15]. These highly evolving genetic elements may also be ideal responsive elements to fluctuating selective pressures. A role in evolutionary selection and adaptation is consistent with deep evolutionary conservation of some STRs, as “tuning knobs”, including several in genes with neurological and neurodevelopmental function[16].

While a limited number of studies indicate that purifying selection and drift can shape the structure of STRs at the inter- and intra-species levels [17–22], the global abundance of STRs at the crossroads of speciation remains largely unknown.

Mononucleotide and dinucleotide STRs are the most common categories of STRs in the vertebrate genomes[23, 24]. In addition to their association with frameshifts in coding sequences and pathological [25] and possibly evolutionary consequences, recent evidence indicates surprising functions for the mononucleotide STRs, such as their proposed role in translation initiation site selection[12, 26]. Several groups have found evidence on the involvement of a number of dinucleotide STRs in gene regulation, speciation, and evolution[4, 23, 27–30]. Trinucleotide STRs are frequently linked to human neurological disorders, most of which are specific to this species[31, 32].

Here, we analyzed the global hierarchical clustering of all types of mono-, di-, and trinucleotide STRs in nine mammalian species, encompassing primates and rodents, Those species belong to the superordinal group of Euarchontoglires [33], and form three distinct and unambiguous phylogenetic < clusters>. The aim of this analysis was to examine whether the global abundance of STRs in the selected species conforms to the phylogenetic < clusters > of the selected species, or not.

## Materials and methods

### Species and whole-genome sequences

The UCSC genome browser (https://hgdownload.soe.ucsc.edu) was used to download and analyze the latest genome assemblies of nine species as follows (genome sizes are indicated following each species): rat (*Rattus norvegicus*): 2,647,915,728, mouse (*Mus musculus*): 2,728,222,451, gelada (*Theropithecus gelada*): 2,889,630,685, olive baboon (*Papio anubis*): 2,869,821,163, macaque (*Macaca mulatta*): 2,946,843,737, gorilla (*Gorilla gorilla gorilla*): 3,063,362,754, chimpanzee (*Pan troglodytes*): 3,050,398,082, bonobo (*Pan paniscus*): 3,203,531,224, and human (*Homo sapiens*): 3,099,706,404. Those species encompassed rodents: rat and mouse, Old World monkeys: gelada, olive baboon, macaque, and great apes: gorilla, bonobo, chimpanzee, human.

### Extraction of STRs from genomic sequences

The whole-genome abundance of mononucleotide STRs of ≥ 10-repeats, dinucleotide STRs of ≥ 6-repeats, and trinucleotide STRs of ≥ 4-repeats were studied in the nine selected species. To that end, we designed a software package in Java (https://github.com/arabfard/Java_STR_Finder). All possibilities of mononucleotide motifs, consisting of A, C, T, and G, all possibilities of dinucleotide motifs, consisting of AC, AG, AT, CA, CG, CT, GA, GC, GT, TA, TC, and TG, and all possibilities of trinucleotide motifs, consisting of AAC, AAT, AAG, ACA, ACC, ACT, ACG, ATA, ATC, ATT, ATG, AGA, AGC, AGT, AGG, CAA, CAC, CAT, CAG, CCA, CCT, CCG, CTA, CTC, CTT, CTG, CGA, CGC, CGT, CGG, TAA, TAC, TAT, TAG, TCA, TCC, TCT, TCG, TTA, TTC, TTG, TGA, TGC, TGT, TGG, GAA, GAC, GAT, GAG, GCA, GCC, GCT, GCG, GTA, GTC, GTT, GTG, GGA, GGC, and GGT were analyzed.

The written program calculated based on perfect (pure) STRs. The algorithm started from an initial point, which was the first nucleotide of each genome, and iteratively repeated a series of steps during walking on the genome, nucleotide by nucleotide. In the first step, it investigated a window frame of 2*N, where 2 was the definition of tandem repeats i.e., two identical continuous sequences, and N was the length of the STR core. If the first half of the sequence inside the window was not equal to the second half, the algorithm moved one nucleotide forward. If equal, the algorithm checked the nucleotides, and this process continued until all identical continuous nucleotides, which were the same as the core were found. The final selected sequence- M*N- was introduced as a new STR, which had a core with a length of N and M repeats. All steps were repeated to find new STRs from the end of the previous STR. We repeated the algorithm for different values of N (N was between 1 and 3 in each genome to detected mono, di, and trinucleotide STRs).

### Whole-genome STR data aggregation, abundance, and hierarchical cluster analysis across species

Whole-genome chromosome-by-chromosome data were aggregated and analyzed in the nine species. STR abundances across the selected species were obtained and depicted by boxplot diagrams and hierarchical clustering, using boxplot and hclust packages[34] in R, respectively. Boxplots illustrate abundance differences among segments across the selected species, and hierarchical clustering plots demonstrate the level of similarity and differences across the obtained abundances. The input data to these packages were numerical arrays . Each array consisted of a number of columns, each column corresponding to the STR abundance in different chromosomes. It should be noted that the focus of our analysis was to evaluate the global abundance of STRs across those species, regardless of the homologous regions.

### Statistical analysis

The STR abundances across the nine selected species were compared by repeated measurements analysis, using one and two-way ANOVA tests. These analyses were confirmed by nonparametric tests.

## Results

### Global abundance of mono, di, and trinucleotide STRs coincides with the phylogenetic distance of the nine selected species

**Fig. 1:**
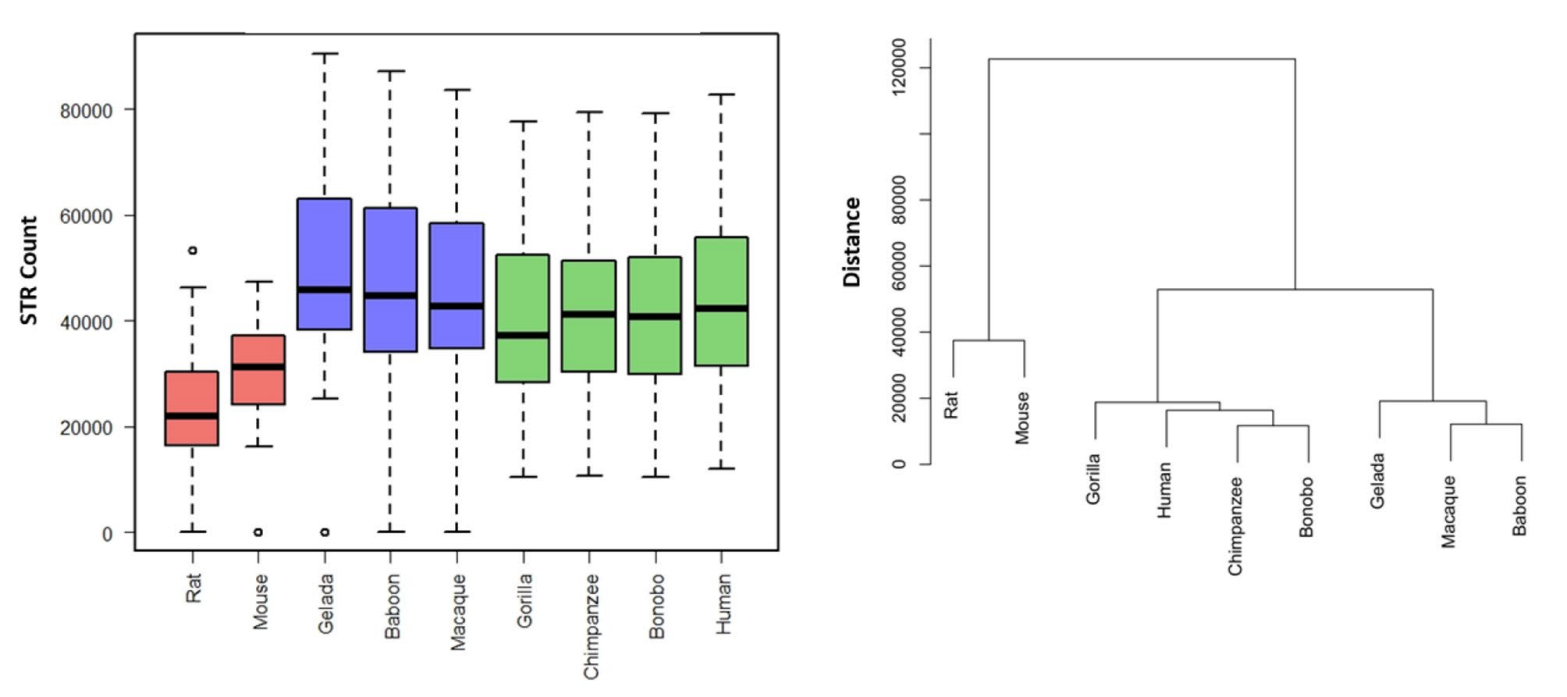
Whole-genome mononucleotide STR abundance in the nine selected species. Global incremented pattern was observed in the primate species versus rodents (left graph). The overall hierarchical clustering yielded three <clusters>, which conformed to <rodents>, <Old World monkeys>, and <great apes> (right graph).

Whole-genome data was collected on the abundance of mononucleotide STRs across the nine species (Table 1). We found massive expansion of the mononucleotide STR compartment in all primate species versus rat and mouse. Hierarchical clustering yielded three < clusters > as follows: <rat, mouse>, <gelada, olive baboon, macaque>, and < gorilla, chimpanzee, bonobo, human>, which coincided with the phylogenetic distance of the nine selected species (P = 6.3E-09) (Fig. 1) namely < rodents>, <Old World monkeys>, and < great apes>.

**Table 1:** Mononucleotide STR abundance across the nine selected species

Chromosome/Species	Rat	Mouse	Gelada	Baboon	Macaque	Gorilla	Chimpanzee	Bonobo	Human
**1**	53,318	47,294	90,549	87,241	83,595	77,718	79,390	79,173	82,820
**2(A)**	46,221	45,636	71,588	67,963	64,609	35,908	35,897	34,400	78,550
**2(B)**	0	0	0	0	0	40,245	39,968	39,837	0
**3**	36,364	38,493	70,736	68,688	65,836	62,398	62,713	64,472	64,027
**4**	34,818	39,019	62,831	60,726	57,817	54,896	54,855	53,287	56,495
**5**	36,532	38,805	66,164	64,101	61,533	60,436	48,944	54,142	56,538
**6**	28,617	35,751	63,104	61,642	59,150	53,872	53,769	53,420	55,185
**7(A)**	29,411	33,649	25,699	65,267	63,438	50,898	53,882	50,792	56,257
**7(B)**	0	0	42,663	0	0	0	0	0	0
**8**	27,353	31,938	50,576	48,446	46,757	43,593	44,212	43,618	45,220
**9**	23,532	31,142	50,050	47,879	46,910	36,797	38,035	37,493	41,744
**10**	31,065	34,138	41,475	39,012	37,477	44,166	44,562	44,416	46,075
**11**	17,071	33,869	54,287	54,284	51,654	37,218	41,059	40,757	42,217
**12**	15,101	29,325	42,675	35,365	42,793	46,865	47,576	47,481	48,483
**13**	21,673	29,496	40,602	39,101	38,022	27,902	28,481	28,479	29,430
**14**	21,835	28,835	45,820	44,693	42,677	30,311	30,659	30,595	31,460
**15**	20,351	25,753	43,334	41,671	40,009	28,611	29,752	29,049	31,402
**16**	15,958	24,139	41,211	39,781	37,693	29,268	31,121	28,460	34,364
**17**	18,458	24,234	32,308	31,285	30,378	29,884	36,791	37,010	38,947
**18**	16,651	22,580	25,310	24,850	23,551	22,556	22,428	22,236	23,130
**19**	14,266	16,221	35,819	32,702	30,470	23,832	31,405	30,614	32,423
**20**	14,475	0	34,962	32,965	32,095	20,654	22,106	31,034	21,961
**21**	0	0	0	0	0	10,462	10,633	10,467	12,050
**22**	0	0	0	0	0	13,778	14,816	13,904	16,014
**X**	25,983	40,547	52,836	49,013	47,590	43,138	43,302	41,656	46,178
**Sum**	**549,053**	**650,864**	**1,084,599**	**1,036,675**	**1,004,054**	**925,406**	**946,356**	**946,792**	**990,970**

**Fig. 2:**
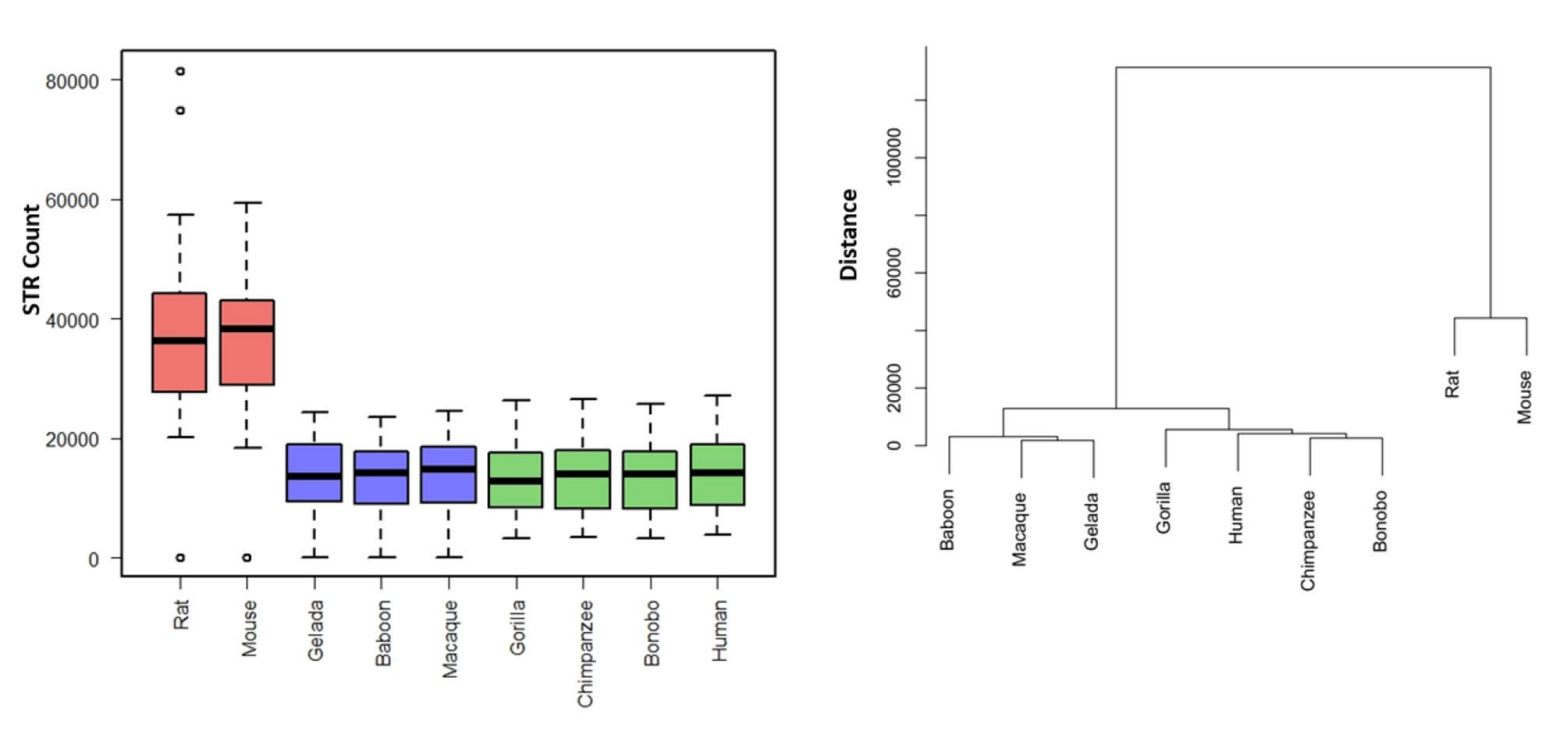
Whole-genome dinucleotide STR abundance in the nine selected species. Global decremented patterns were observed in all primate species versus mouse and rat (left gragh). The global pattern conformed to the three <clusters> across the nine species and their phylogenetic distance (right graph)

The whole-genome STR abundances from aggregated chromosome-by-chromosome analysis in the dinucleotide category (Table 2) was decremented in primates versus rodents. Similar to the mononucleotide STR compartment, the dinucleotide STR compartment conformed to the genetic distance among the three < clusters > of species (P = 7.1E-08) (Fig. 2).

**Table 2:** Dinucleotide STR abundance across the nine selected species

Chromosome/Species	Rat	Mouse	Gelada	Baboon	Macaque	Gorilla	Chimpanzee	Bonobo	Human
**1**	81,509	59,425	24,335	23,427	24,462	23,105	23,708	23,583	24,657
**2(A)**	74,837	53,096	21,315	20,302	21,225	11,820	11,960	11,391	26,989
**2(B)**	0	0	0	0	0	14,494	14,555	14,334	0
**3**	53,642	45,464	20,710	19,973	20,552	20,939	21,179	21,039	21,633
**4**	57,299	44,963	19,364	18,592	19,038	21,536	21,182	20,503	21,773
**5**	52,269	48,069	22,020	21,275	22,147	17,099	17,831	19,606	20,385
**6**	44,993	45,325	19,921	19,397	20,070	18,575	18,391	18,196	18,995
**7(A)**	43,219	40,052	5832	16,963	17,870	15,988	16,727	16,130	17,275
**7(B)**	0	0	11,934	0	0	0	0	0	0
**8**	43,242	41,103	15,903	15,390	16,164	15,837	15,875	15,718	16,245
**9**	37,463	39,005	14,733	14,183	14,857	11,704	11,935	11,661	13,080
**10**	40,260	40,998	10,136	9432	9855	14,051	14,306	14,032	14,799
**11**	27,685	38,212	14,360	14,487	15,187	12,678	13,988	13,842	14,189
**12**	22,084	35,361	13,478	14,325	14,685	14,385	14,559	14,588	14,757
**13**	38,331	35,159	11,839	11,292	11,797	11,071	11,258	11,135	11,406
**14**	31,923	36,644	13,605	13,243	13,885	9549	9465	9386	9798
**15**	31,768	30,662	12,078	11,661	12,014	8014	8226	8143	8607
**16**	28,704	29,521	8228	8064	8206	7814	8268	7553	8947
**17**	30,312	28,209	11,002	10,457	10,942	10,456	8056	8006	8355
**18**	27,797	27,263	8548	8349	8591	8629	8597	8497	8750
**19**	21,794	18,350	5994	5493	5395	4774	6081	5865	6220
**20**	20,191	0	8334	7902	8345	6379	7106	6623	6612
**21**	0	0	0	0	0	4092	4154	4123	4884
**22**	0	0	0	0	0	3209	3442	3183	3746
**X**	36,246	38,470	18,303	16,787	17,659	17,922	18,193	17,078	18,952
**Sum**	**845,568**	**775,351**	**311,972**	**300,994**	**312,946**	**304,120**	**309,042**	**304,215**	**321,054**

**Fig. 3:**
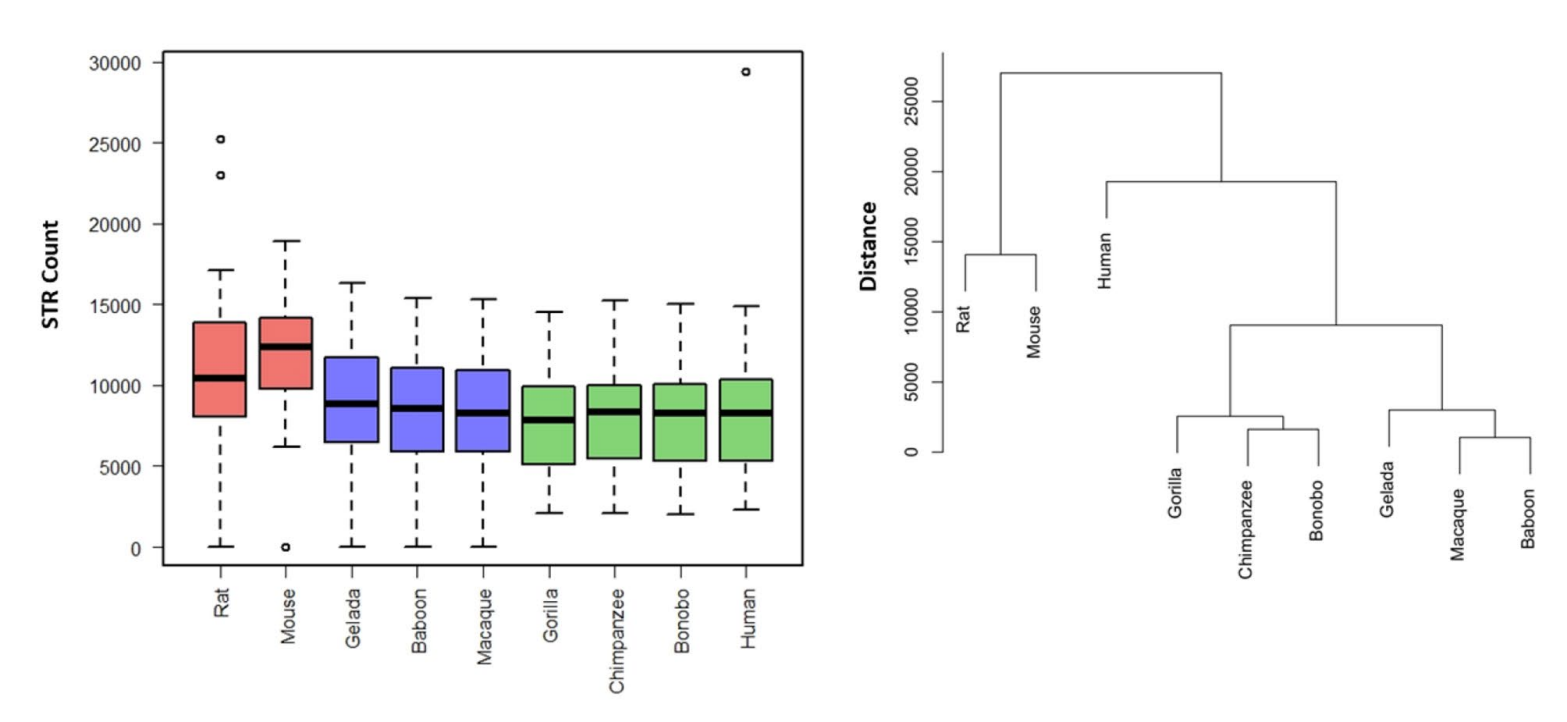
Whole-genome trinucleotide STR abundance in the nine selected species. While global decremented patterns were observed in primates versus rodents (left graph), human stood out in this category, in comparison to all other species (right graph)

There was global shrinkage of the trinucleotide STR compartment in primates versus rodents (P = 3.8E-05) (Table 3; Fig. 3). Remarkably, human stood out among all other species in the trinucleotide STR compartment.

**Table 3:** Trinucleotide STR abundance across the nine selected species

Chromosome/Species	Rat	Mouse	Gelada	Baboon	Macaque	Gorilla	Chimpanzee	Bonobo	Human
**1**	25,234	18,913	16,307	15,350	15,341	14,540	15,219	15,054	14,882
**2(A)**	22,996	17,856	13,005	12,341	11,998	6800	6842	6537	14,521
**2(B)**	0	0	0	0	0	7545	7764	7822	0
**3**	16,869	15,022	12,749	12,518	11,938	11,473	11,744	11,637	11,631
**4**	17,088	15,204	11,921	11,154	10,960	11,116	11,228	10,685	11,144
**5**	16,339	15,469	13,001	12,514	12,112	10,581	9665	10,640	10,649
**6**	13,495	14,332	12,150	11,743	11,380	10,364	10,504	10,445	29,430
**7(A)**	14,317	13,760	3937	10,991	10,871	9342	10,117	9744	9995
**7(B)**	0	0	7552	0	0	0	0	0	0
**8**	12,701	13,518	10,032	9524	9682	8752	9096	8645	8890
**9**	11,646	12,378	9295	8755	8659	6898	7328	7157	7580
**10**	12,552	13,968	7297	6728	6786	8096	8350	8245	8295
**11**	7987	13,232	9615	9578	9403	7801	8668	8458	8352
**12**	6060	11,817	7742	8297	8029	8905	9218	9051	9127
**13**	10,852	11,634	7266	6823	6860	5273	5479	5452	5391
**14**	10,325	11,865	8869	8583	8253	5473	5771	5785	5706
**15**	10,075	10,693	7727	7339	7152	4869	5168	5082	5297
**16**	8476	9527	6228	5837	5801	5738	6007	5623	6402
**17**	9502	10,045	5908	5737	5684	5666	5859	5914	6091
**18**	8124	9154	4738	4645	4603	4722	4625	4584	4566
**19**	6984	6190	5432	4643	4664	3807	5438	5230	5101
**20**	6445	0	6655	6016	5945	4072	4472	4155	4130
**21**	0	0	0	0	0	2051	2092	2028	2304
**22**	0	0	0	0	0	2721	2825	2601	2915
**X**	10,411	13,783	11,449	10,609	10,666	9547	9838	9140	10,062
**Sum**	**258,478**	**258,360**	**198,875**	**189,725**	**186,787**	**176,152**	**183,317**	**179,714**	**202,461**

### Differential abundance patterns of various STRs and STR lengths across rodents and primates

**Fig. 4:**
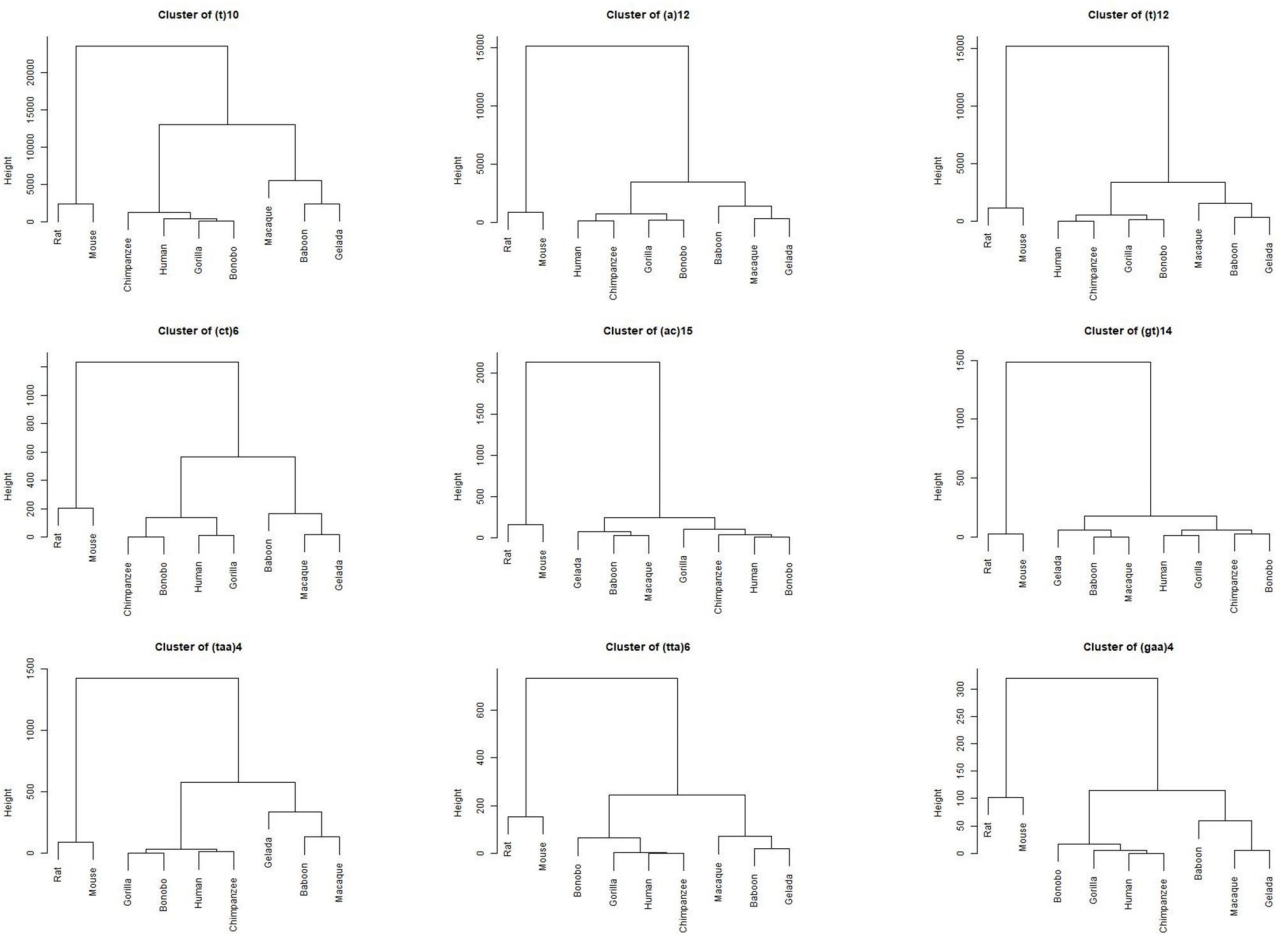
Example of STRs and STR lengths, abundance of which coincided with the phylogeny of the nine selected species. Three STRs are depicted as examples for each of mono, di, and trinucleotide categories. Data from all studied STRs are available at: https://figshare.com/articles/figure/STR_Clustering/17054972

Numerous STRs and STR lengths across the mono, di, and trinucleotide STR categories conformed to the phylogenetic distances of the nine selected species, for example, in the instance of T/A mononucleotides of 10, 11, and 12 repeats, which were the most abundant STRs across all nine species (Fig. 4). In another example, (ct)6 and (taa)4 conformed to the phylogeny of the studied species in the di and trinucleotide STR categories, respectively.

**Fig. 5:**
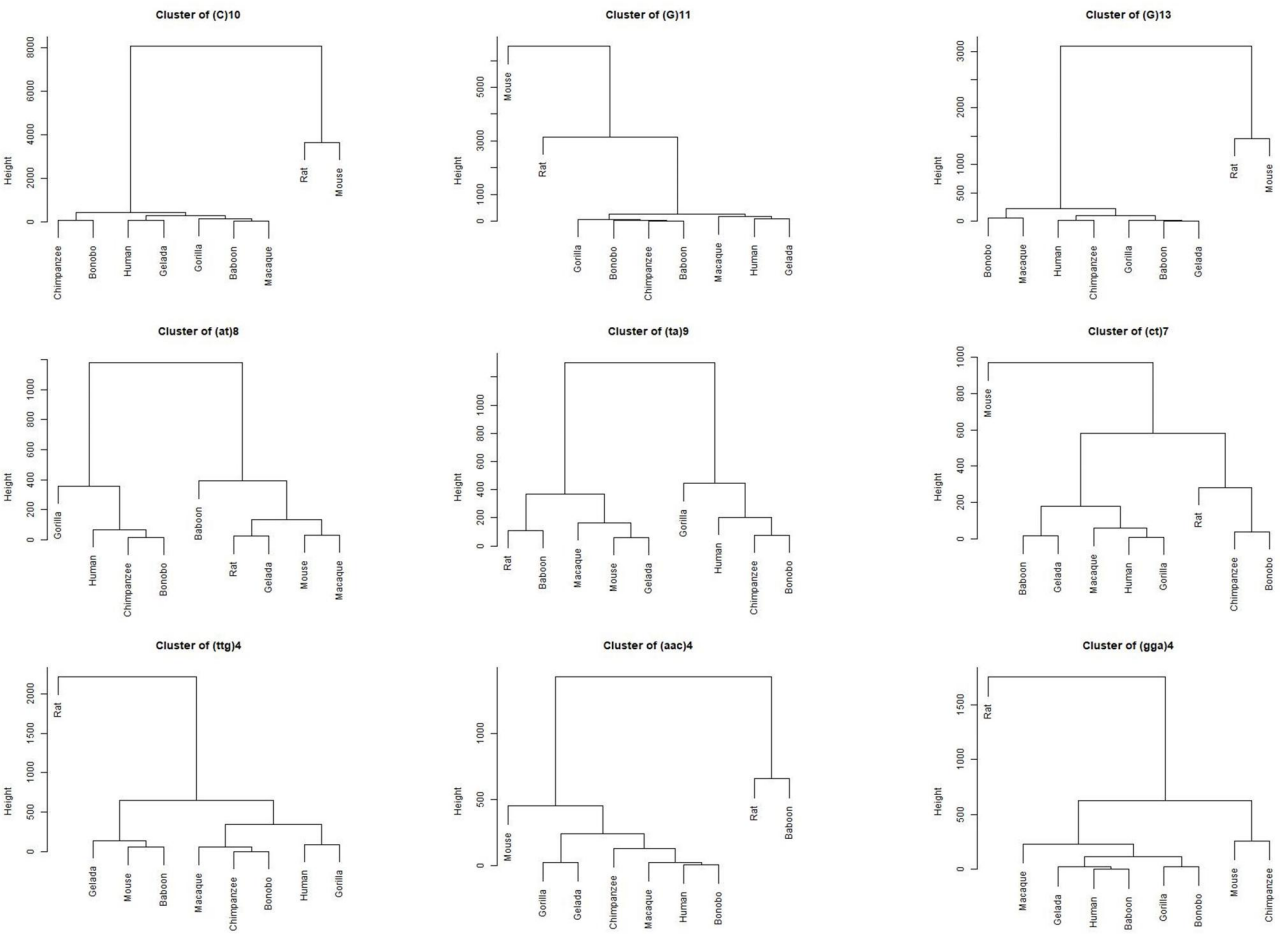
Example of STRs and STR lengths, abundance of which appeared to be predominantly random across the nine selected species. Three STRs are depicted as examples for each of mono, di, and trinucleotide categories. Data from all studied STRs are available at: https://figshare.com/articles/figure/STR_Clustering/17054972

On the other hand, numerous STRs did not follow perfect phylogenetic patterns, such as (C)10, (at)8, and (ttg)4 (Fig. 5). Hierarchical clusters of all studied STRs across the three categories are available at: https://figshare.com/articles/figure/STR_Clustering/17054972.

## Discussion

While the mechanisms underlying speciation are extremely complicated and largely based on theories and models, the impact of genetics seems to be significant in respect of adaptation, gene flow, and natural selection. In fact, natural selection may be a central converging point of the evolutionary propositions for speciation. However, the various mechanisms involved in speciation have different impact on natural selection, and it is the net effect which may ultimately result in the emergence of a new species.

As one of the most abundant genetic elements in various animal genomes, it is largely unknown whether at the crossroads of speciation, STRs evolved as a result of purifying selection, genetic drift, and/or in a directional manner.

Here, we selected multiple species across rodents and primates, and investigated the clustering patterns of all possible types and lengths of mononucleotides, dinucleotide, and trinucleotide STRs on the whole-genome scale in those species. Hierarchical clustering yielded clusters that predominantly conformed to the phylogenetic distances of the selected species. Hierarchical clustering is an unsupervised clustering method that is used to group data. This algorithm is unsupervised because it uses random, unlabeled datasets. As the number of clusters increases, the accuracy of the hierarchical clustering algorithm improves.

**Fig. 6:**
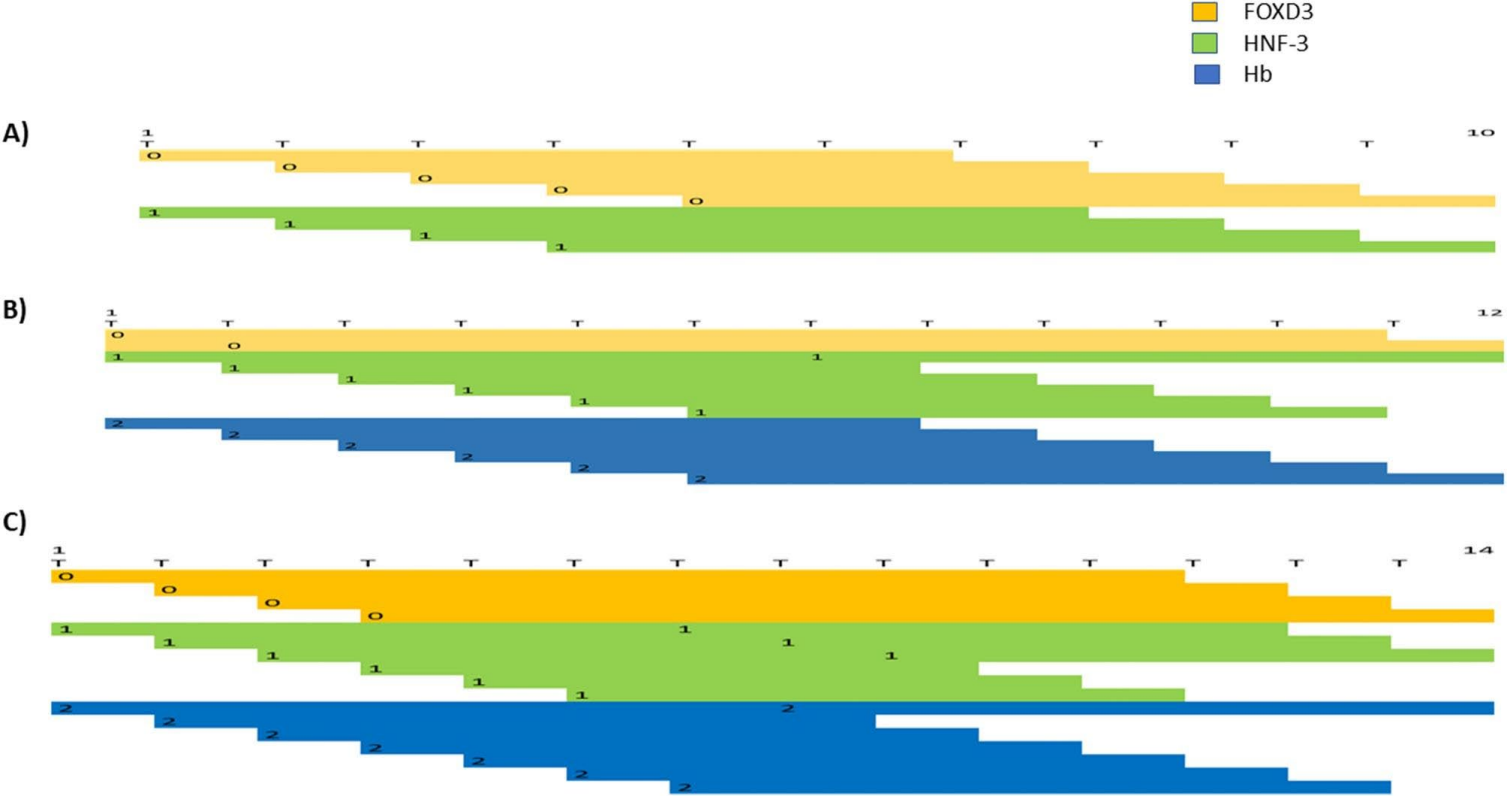
Potential recruitment of qualitatively and quantitatively different TFs to various lengths of (T)-repeats. (T)10 (**A**) and (T)12 (**B**) conformed to the phylogenetic < clusters>, whereas (T)14 (**C**) did not. Differential recruitment of TFs may differentially regulate the relevant genes in evolutionary processes

**Fig. 7:**
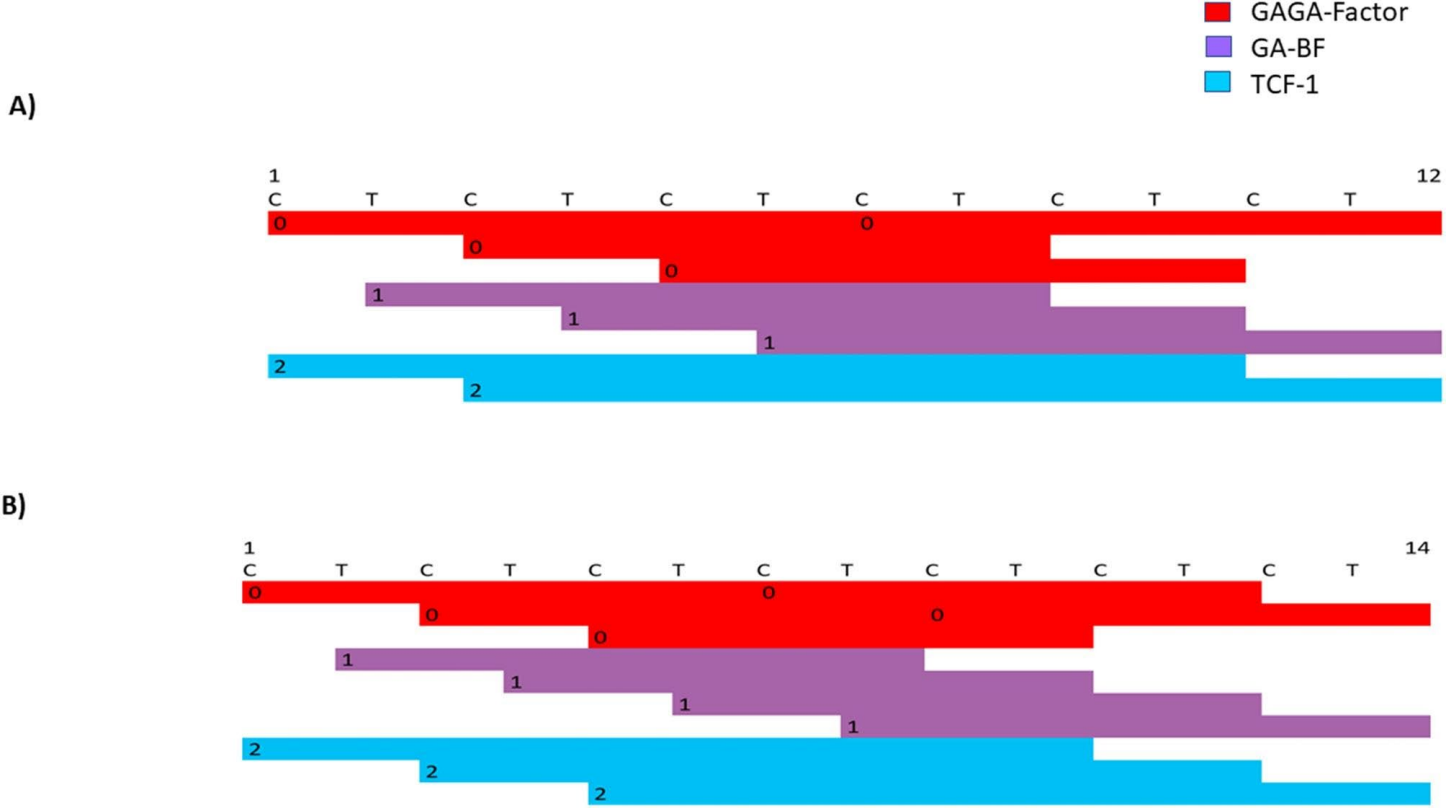
Potential differential TF recruitments to various lengths of (ct)6 **A**) and (ct)7 **B**). Those two lengths result in alternative quantitative binding of three TFs. (ct)6 conformed and (ct)7 did not conform to the phylogenetic < clusters>

Our findings may be of significance in a number of aspects. Firstly, there were significant differential abundances separating rodents from primates, for example, massive decremented abundance of dinucleotide and trinucleotide STRs in primates versus the rodent species, and massive incremented abundance of mononucleotide STRs in primates versus rodents. Secondly, the three major < clusters > obtained from global hierarchical cluster analysis matched the phylogeny of the three < clusters > of species, i.e., <rodents>, <Old World monkeys>, and < great apes>. It is possible that there are mathematical channels/thresholds required for the abundance of STRs in various orders. This is in line with the hypothesis that STRs function as scaffolds for biological computers[35]. In addition, our data indicate that various STRs and STR lengths behave differently with respect to their colossal abundance. Not all the studied STRs conformed to the phylogenetic distances of the nine selected species. We hypothesize that those which did, had a link with the speciation of those species, whereas those which did not, apparently followed random patterns for the most part. The potential effect of STRs in non-genic regions is largely unknown. However, when located at genic regions, various STRs and repeat lengths can potentially recruit transcription factors (TFs), which differ in qualitative and quantitative terms (http://alggen.lsi.upc.es/cgi-bin/promo_v3/promo/promoinit.cgi?dirDB=TF_8.3) [36]. Those various TF sets may differentially regulate expression of the relevant genes during the process of evolution. For example, T-blocks of 10, 12, and 14-repeats recruit various combinations of FOXD3, HNF-3, and Hb (Fig. 6). Interestingly, (T)10 and (T)12 were among the mononucleotide STRs, which conformed to the phylogenetic distance of the nine species (Fig. 4), and (t)14 did not (https://figshare.com/articles/figure/STR_Clustering/17054972). The concept of various TF sets stands for other STRs as well. For example, (ct)6 conforms to the phylogenetic clusters, and recruits a number of TFs, whereas (ct)7, which does not conform to those clusters, recruits quantitatively different set of those TFs (Fig. 7).

Mononucleotide STRs impact various processes, such as gene expression, translation alterations, and frameshifts of various proteins, which may have evolutionary and pathological consequences[12, 25]. They can overlap with G4 structures, many of which associate with evolutionary consequences[37].

In a number of instances, dinucleotide STRs located in the protein-coding gene core promoters have been subject to contraction in the process of human and non-human primate evolution[38]. A number of those STRs are identical in formula in primates versus non-primates, and the genes linked to those STRs are involved in characteristics that have diverged primates from other mammals, such as craniofacial development, neurogenesis, and spine morphogenesis. Structural variants are enriched near genes that diverged in expression across great apes[39], and genes with STRs in their regulatory regions are more divergent in expression than genes with fixed or no STRs[40]. STR variants are likely to have epistatic interactions, which can have significant consequences in complex traits, in human as well as model organisms[6, 41].

Trinucleotide STRs are predominantly focused on in human because of their link with several neurological disorders[42–45]. We found an exceptional global hierarchical distance between human and all other species in that compartment. In view of the fact that most of the phenotypes attributed to trinucleotide STRs are human-specific in nature, it is conceivable that their evolution is also significantly distant from all other species studied.

The observed abundances were independent of the genome sizes of the selected species. For example in the instances of di- and trinucleotide STRs, we observed higher abundances in rodents versus primates despite the smaller genome sizes of the former. These findings are in line with the previous reports of lack of relationship between genome size and abundance of STRs[46, 47].

It should be noted that this is a pilot study based on hierarchical clustering, and future studies are warranted to further examine our hypothesis, using phylogenetic platforms and additional orders and species. Functional studies are also warranted to examine the biological impact of the relevant STRs.

## Conclusion

We propose that the global abundance of STRs is non-random across rodents and primates. We also propose the STRs and STR lengths, which predominantly conformed to the phylogenetic distances of those species, such as (t)10, (ct)6, and (taa4). Additional species encompassing other orders and phylogenetic platforms are warranted to further examine this proposition.

## Limitations

This research was a pilot study based on hierarchical clustering of the collected data in a number of mammalian species. Phylogenetic platforms and additional orders of species are warranted to further examine our hypothesis.

## Data Availability

Raw data are available at: https://figshare.com/articles/dataset/Trends/15073329 and https://figshare.com/articles/figure/STR_Clustering/17054972.

## Abbreviations

STR: Short tandem repeat
TF: Transcription factor
